# Atlantic cod (*Gadus morhua*) hemoglobin genes: multiplicity and polymorphism

**DOI:** 10.1186/1471-2156-10-51

**Published:** 2009-09-03

**Authors:** Tudor Borza, Cynthia Stone, A Kurt Gamperl, Sharen Bowman

**Affiliations:** 1 Genome Atlantic, NRC Institute for Marine Biosciences, 1411 Oxford Street, Halifax, NS, B3H 3Z1, Canada; 2 Ocean Sciences Centre, Memorial University of Newfoundland, St. John's, NL, A1C 5S7 Canada

## Abstract

**Background:**

Hemoglobin (Hb) polymorphism, assessed by protein gel electrophoresis, has been used almost exclusively to characterize the genetic structure of Atlantic cod (*Gadus morhua*) populations and to establish correlations with phenotypic traits such as Hb oxygen binding capacity, temperature tolerance and growth characteristics. The genetic system used to explain the results of gel electrophoresis entails the presence of one polymorphic locus with two major alleles (HbI-1; HbI-2). However, vertebrates have more than one gene encoding Hbs and recent studies have reported that more than one Hb gene is present in Atlantic cod. These observations prompted us to re-evaluate the number of Hb genes expressed in Atlantic cod, and to perform an in depth search for polymorphisms that might produce relevant phenotypes for breeding programs.

**Results:**

Analysis of Expressed Sequence Tags (ESTs) led to the identification of nine distinct Hb transcripts; four corresponding to the α Hb gene family and five to the β Hb gene family. To gain insights about the Hb genes encoding these transcripts, genomic sequence data was generated from heterozygous (HbI-1/2) parents and fifteen progeny; five of each HbI type, i.e., HbI-1/1, HbI-1/2 and HbI-2/2. β Hb genes displayed more polymorphism than α Hb genes. Two major allele types (β1A and β1B) that differ by two linked non-synonymous substitutions (Met55Val and Lys62Ala) were found in the β1 Hb gene, and the distribution of these β1A and β1B alleles among individuals was congruent with that of the HbI-1 and HbI-2 alleles determined by protein gel electrophoresis. RT-PCR and Q-PCR analysis of the nine Hb genes indicates that all genes are expressed in adult fish, but their level of expression varies greatly; higher expression of almost all Hb genes was found in individuals displaying the HbI-2/2 electrophoretic type.

**Conclusion:**

This study indicates that more Hb genes are present and expressed in adult Atlantic cod than previously documented. Our finding that nine Hb genes are expressed simultaneously in adult fish suggests that Atlantic cod, similarly to fish such as rainbow trout, carp, and goldfish, might be able to respond to environmental challenges such as chronic hypoxia or long-term changes in temperature by altering the level of expression of these genes. In this context, the role of the non-conservative substitution Lys62Ala found in the β1 Hb gene, which appears to explain the occurrence of the HbI-1 and HbI-2 alleles described by gel electrophoresis, and which was found to be present in other fish such as eel, emerald rockcod, rainbow trout and moray, requires further investigation.

## Background

Hemoglobin (Hb) polymorphisms, identified by means of protein gel electrophoresis, have been used for decades to assess the genetic structure of populations, to establish relationships between genotypes and phenotypes, and to understand the molecular bases of genetic diseases [1-3].

The Atlantic cod (*Gadus morhua*) represents one of the most valuable commercial resources for fisheries in the northern Atlantic, contributing to the cultural identity of people living in the costal areas of Atlantic Canada, Iceland and Northern Europe. In recent years, due to decline in the worldwide catch and to the increase in the retail price, Atlantic cod has become an attractive species for aquaculture [4-6]. As a result of its economic importance, many studies have investigated the relationship between the Hb types revealed by protein electrophoresis and phenotypic traits such as Hb oxygen binding capacity, temperature tolerance and growth characteristics. Almost five decades ago, Sick [7] demonstrated that Atlantic cod Hbs can be separated by agar gel electrophoresis into two main components, HbI and HbII, the first component showing three different variants, HbI-1/1, HbI-1/2 and HbI-2/2. The genetic model put forward to explain these polymorphisms was a two-allele system, with subsequent studies suggesting that the distribution of Hb alleles in Atlantic cod populations is strongly influenced by water temperature; the HbI-1 allele is dominant in warmer regions (North Sea, southern part of the Norwegian Sea, western part of the Baltic Sea and around the British Isles), while allele HbI-2 is predominantly found in colder waters (Greenland, Iceland, Canada, northern Norway and the northern part of the Baltic Sea) [8-10]. North-south clines in HbI allele frequencies were identified in the eastern North Atlantic [3,8-11]. However, the existence of these clines in the western North Atlantic was less conspicuous [3,11,12]. Karpov and Novikov [13] provided a possible physiological basis for this distribution by showing that the temperature effect on *in vitro *hemoglobin-oxygen dissociation curves differed substantially between the three common HbI genotypes, a finding supported by other, more recent, studies [14,15]. These studies show that the affinity of hemoglobin for oxygen is higher for HbI-2/2 individuals at low temperatures (<10°C) [13-15], that hemoglobin-oxygen binding affinity becomes greater for HbI-1/1 individuals at high temperatures (>14°C) [13-15] and that the heterozygous hemoglobin type has oxygen affinity values that are intermediate between HbI-1/1 and HbI-2/2 [13]. There are many other reports on the apparent selection effects on the HbI types, including the age and seasonality of sexual maturation [16], annual mortality [17], haematocrit [18] and number and diameter of muscle fibers [19]. Further, it is apparent that hemoglobin components undergo changes during ontogeny and in response to various stimuli. For instance, Hb electrophoresis indicates that in juveniles the apparently monomorphic HbII components are more visible than HbI while in adult fish the main components are represented by the polymorphic HbI [20]. Also, acclimation to lower (4 and 8°C) and higher (12 and 15°C) temperatures favours the expression of more anodic and cathodic hemoglobin components, respectively [14].

However, research on Atlantic cod hemoglobin polymorphism has not always produced results that are consistent with the apparent relationship between water temperature and hemoglobin-oxygen binding characteristics. For example, while Petersen and Steffensen [15] showed that hemoglobin genotype correlated well with temperature preference (juvenile HbI-2/2 Atlantic cod preferring a temperature of 8.2°C while HbI-1/1 preferred 15.4°C), Gamperl et al. [21] were unable to demonstrate that upper temperature or hypoxia tolerance differed between hemoglobin genotypes when acutely exposed to these environmental challenges. In addition, although most growth studies show that HbI-2/2 Atlantic cod have the highest overall growth of the three Hb types [22,23], and that temperature-related differences in growth among genotypes are photoperiod dependent [24], the temperature at which Atlantic cod of all three variants was reported to be growing best varies widely between studies, e.g., 12°C [14], 14°C [25] or between 12.5°C and 14.5°C [23]. Discrepancies among the results of these studies might be due to the fact that protein electrophoresis largely underestimates genetic variability [26] and that many polymorphisms present in Hb molecules are not detected using this method. Moreover, the heterotetrameric hemoglobin molecule of fish consists of two α-globin subunits and two β-globin subunits that are encoded by different genes, most of them being polymorphic [27,28], as seen in other vertebrates. Indeed, more Hb variants have been described in Atlantic cod after using more refined methods such as protein isoelectrofocusing [14,29], Hb purification by chromatography followed by amino acid sequencing [30], and the characterization of hemoglobin single nucleotide polymorphisms (SNPs) using limited sequence information [31]. However, no in depth genomic studies of the Hb genes that are expressed in Atlantic cod have been performed to date.

Canadian and Norwegian Atlantic cod genomics projects have generated a large amount of sequence data [[6], Bowman et al, in prep]. The Cod Genomics and Broodstock Development Project (CGP) recently produced 158,877 expressed sequence tags (ESTs) [Bowman et al, in prep] using a large number of individuals and a variety of cDNA libraries, including several blood, embryonic and larval cDNA libraries. We decided to use this information to: 1) to determine how many different Hb transcripts are present in Atlantic cod; 2) find out if specific Hb transcripts are expressed in discrete developmental stages and 3) uncover polymorphisms present in the Hb transcripts. To gain more information about the Hb genes present in Atlantic cod we also generated genomic sequence data from fifteen individuals, five of each HbI type, i.e., HbI-1/1, HbI-1/2 and HbI-2/2, from a family produced by crossing heterozygous parents (both HbI-1/2), and determined the expression of individual Hb genes by quantitative PCR (Q-PCR).

## Results

### Identification of different Hb transcripts

The search performed in the CGP database , which comprises 160,228 Atlantic cod ESTs, led to the identification of several clusters annotated by AutoFact [32] as corresponding to Hb sequences. The ESTs from these clusters were pooled with homologous EST sequences retrieved from GenBank databases and re-clustered. TBLASTX search in GenBank (survey performed in June, 2008) revealed that approximately 1% of the 186,753 Atlantic cod ESTs deposited in GenBank by CGP and other submitters correspond to different Hb transcripts. The final clustering of these ESTs produced 4 α-like and 5 β-like unique sequences (contigs). Multiple polymorphisms were evident in the contigs that resulted from clustering the β Hb ESTs, suggesting that some of these contigs might contain ESTs that are the product of more than one gene, or that they correspond to multiple alleles of the same gene.

### Genomic data for the Atlantic cod Hb genes

To gain more information about the Hb genes present in Atlantic cod and about the polymorphisms associated with these genes, genomic data was produced by sequencing amplicons generated by PCR from five individuals of each Hb type, i.e., HbI-1/1, HbI-1/2, and HbI-2/2, and from their heterozygous parents (HbI-1/2)(for more details on this family see the Methods section). Good quality sequence data was obtained for the four α Hb genes; in contrast, high quality data was obtained only for two (Hb β2 and β5) of the five β Hb genes found in Atlantic cod. The chromatograms resulting from the sequencing of the Hb genes β1, β3 and β4 were of poor quality or difficult to decipher. Analysis of these chromatograms revealed numerous frame-shifting indels in intronic sequences, as well as a large number of SNPs, suggesting the presence of more than one allele/locus in each individual analysed. For this reason, the PCR products of these β Hb genes had to be cloned before sequencing.

Atlantic cod Hb genes show an archetypal structure for Hbs, having three exons and two introns. All introns display a standard GT-AC structure at the exon-intron junction and their position in the coding regions is rather similar; however, the length of introns varies from gene to gene. Exceptionally long introns were found in the α3 Hb gene (568 nt and 794 nt for intron 1 and 2, respectively) while intron 2 of the β1 Hb gene contains a GT module repeated up to 60 times.

### Atlantic cod α1-α4, β2 and β5 Hb genes, analysed by direct sequencing of PCR products, show little or no polymorphism in their coding sequence

However, apart from the α2 Hb gene (FJ666966), which appears to be monomorphic, all the other α Hb genes display substitutions or indels affecting intronic sequences. In the α1 Hb gene, two linked SNPs have been identified in intron 1, yielding two alleles (GenBank acc. # FJ666964 and FJ666965). Analysis of the α3 Hb gene revealed two SNPs in intron 2 producing 3 alleles, i.e. allele 1, 1165C/1185A (FJ666967), allele 2, 1165C/1185T (FJ666968), and allele 3, 1165T/1185T (FJ666969), respectively. In addition to these polymorphisms and large intronic sequences, this gene has other peculiar features: it encodes a polypeptide that is only 141 amino acids long while most α Hb genes in fish and other vertebrates encode products consisting of 143 amino acids, and, relative to the other Atlantic cod α Hb sequences, it shows less sequence conservation. Finally, two alleles (FJ666970 and FJ666971) were identified following the analysis of several α4 Hb sequences; the difference between the two alleles is a three nucleotide indel in intron 2.

The β2 (FJ666980 and FJ666981) and β5 Hb genes (FJ666989 and FJ66690), for which genomic data was obtained by sequencing PCR products, showed low levels of polymorphism when compared to other Atlantic cod β Hb genes. The polymorphisms found in β2 and in β5 Hb genes consisted of a SNP located in exon 2 resulting in a non-synonymous substitution, and a SNP located in intron 2, respectively. The distribution of these α and β Hb alleles within the family analysed in this study is summarized in Table 1.

**Table 1:** HbI electrophoretic phenotype, and the different alleles found from the analysis of the nine Hb genes in the 15 progeny and their HbI heterozygote parents.

**Individual** **/Gene**	**Electrophoretic** ** phenotype**	**α1**	**α2**	**α3**	**α4**	**β1**	**β2**	**β3**	**β4**	**β5**
1	HbI-1/1	1/1	M	1/3	1/1	A1/A2	1/-	2/-	1/-	1/1
2	HbI-1/1	1/1	M	1/1	1/1	A1/A2	1/-	-/3	2/-	1/1
3	HbI-1/1	1/1	M	1/1	1/1	A1/A2	1/-	1/-	-/4	1/1
4	HbI-1/1	1/1	M	-	-	A1/A2	2/-	1/-	-/4	-
5	HbI-1/1	1/1	M	-	-	A1/A2	2/-	2/-	1/-	-
6	HbI-1/2	1/1	M	1/3	1/1	A1/B2	1/-	2/-	1/-	1/2
7	HbI-1/2	1/2	M	2/3	1/2	A2/B1	1/2	1/-	-/3	1/2
8	HbI-1/2	1/1	M	2/3	1/2	A1/B2	1/-	2/-	1/-	1/2
9	HbI-1/2	1/2	M	-	-	A2/B1	2/-	1/2	1/-	-
10	HbI-1/2	1/1	M	-	-	A1/B2	1/-	1/-	1/-	-
11	HbI-2/2	1/2	M	-	-	B1/B2	1/-	2/-	1/-	-
12	HbI-2/2	1/2	M	1/3	1/2	B1/B2	1/-	2/-	1/-	2/2
13	HbI-2/2	1/2	M	-	1/2	B1/B2	1/-	2/-	1/-	2/2
14	HbI-2/2	1/2	M	1/3	1/2	B1/B2	2/-	2/-	1/-	2/2
15	HbI-2/2	1/2	M	-	-	B1/B2	1/-	1/-	1/-	-
♂	HbI-1/2	1/1	M	-	-	A2/B2	2/-	1/3	1/3	-
♀	HbI-1/2	1/2	M	-	-	A1/B1	1/-	2/-	2/4	-

Note: The HbI phenotype was determined by Hb electrophoresis on agar gels and by IEF, while genomic data was obtained from sequencing the nine Hb genes. Transcript sequence data was produced only for the β1, β3 and β4 Hb genes. GenBank accession numbers for these genes/alleles are FJ666964 to FJ666990. M, monomorphic locus; -, data not available/not determined

### Analysis of β1, β3 and β4 Hb genes was performed using cloned PCR products as results indicated that frame-shifting polymorphisms were present in these genes

Analysis of the cloned products of the β1 Hb gene revealed multiple SNPs at synonymous sites and three SNPs at non-synonymous sites, as well as many SNPs and indels in intronic sequences, indicating that four alleles for this gene were present in this family, and that both progeny and parents were heterozygotes (Table 1 and Additional file 1). The identification of two non-synonymous substitutions Met55Val and Lys62Ala, linked to several distinct silent substitutions, suggested the presence of two main allele types (haplotypes), β1A and β1B (β1A - Met55/Lys62 and β1B - Val55/Ala62). Each of these main allele types appear to be represented by two subtypes, i.e., β1A1, β1A2 and β1B1, β1B2, respectively, which differ from each other by exhibiting dissimilar polymorphisms at synonymous sites and intronic sequences. The third non-synonymous substitution, Leu123Met, occurs only in the main β1B allele type (β1B1 Met123, and β1B2 Leu123)(FJ666974 and FJ666975, respectively) while the β1A type shows no polymorphism at this position; i.e. β1A alleles retain a Leu in position 123 (FJ666972 and FJ666973). A small number of cloned β1 Hb sequences (FJ666976-FJ666979), identified in both parents and progeny, suggest that some additional β1-like sequences might be present in Atlantic cod and that these sequences likely occurred by gene rearrangements involving exon and/or intron shuffling among alleles (Additional file 1). The distribution of these β1A and β1B alleles among individuals was congruent with that of the HbI alleles determined by Hb gel electrophoresis: i.e the HbI-1/1 individuals displayed only alleles of the main β1A type, the HbI-1/2 individuals alleles of the both types, and the HbI-2/2 individuals alleles of the β1B type (Table 1).

**Figure 1:**
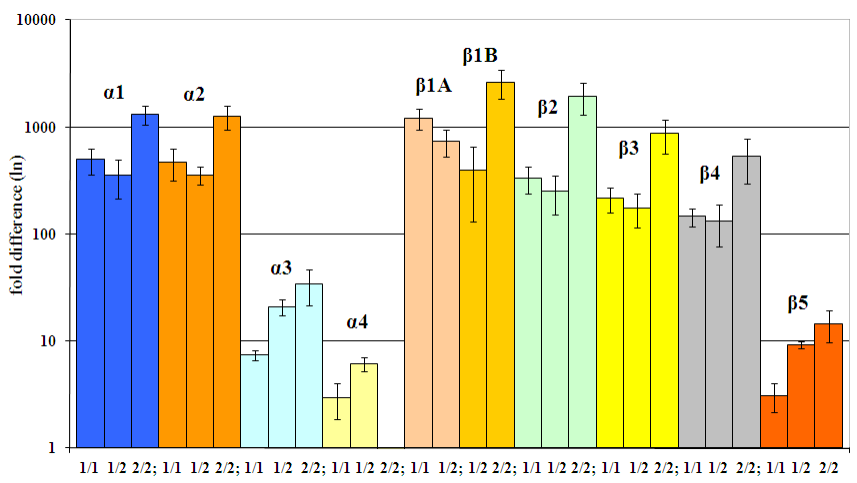
**The expression of the nine Hb genes in individuals of the three HbI electrophoretic types**. Q-PCR analysis was performed using the ΔΔCt method and ubiquitin as a reporter gene. Data for every gene and HbI type was normalized relative to the gene and HbI type with the lowest expression (i.e. the α4 Hb gene, the sample representing the HbI electrophoretic type 2/2).

The data for the β3 and β4 Hb genes was acquired using the same pair of primers (Additional file 2), since the EST data we used for primer design did not allow us to determine whether polymorphisms originated from one locus with multiple alleles or from duplicated loci. Genomic sequence data revealed 12 SNPs and an indel of 4 nucleotides in intron 1, and indicated the presence of 7 distinct alleles in progeny and parents. Phylogenetic study of these alleles (data not shown), together with genotyping analysis of four of these SNPs (Additional file 3) on a large number of individuals from different populations (data not shown), also gave evidence that at least two different loci are required to encode these alleles. The two Hb genes were labelled β3 and β4, with 3 and 4 alleles, respectively (Table 1 and Additional file 3). The sequencing of more clones from every individual and of RT-PCR products, as well as Q-PCR experiments conducted with β3- and β4-specific primers (Figure 1, Table 1 and Additional files 3, 4 and 5), provided additional support for this hypothesis.

### RT-PCR experiments demonstrate that all nine Hb genes are expressed in adult fish

RT-PCR products were obtained for all Hb genes identified in Atlantic cod after 35 cycles of amplification using the total RNA isolated from progeny of the three different HbI types. No information about allele-specific expression could be obtained for the α Hb genes and for β5 as the polymorphisms identified in these genes are located in intronic sequences. To determine if all of the alleles found in the β1-β4 genes are expressed, we sequenced the RT-PCR products generated from the progeny. Transcripts corresponding to all β2-β4 Hb alleles were identified, while the β1 Hb gene data indicated that four alleles are expressed (the sequences corresponding to the GenBank entries FJ666972 to FJ666975). Evidence for a recombinant transcript corresponding to GenBank FJ666976 (Additional file 1), could not be found.

### Q-PCR analysis indicates gene- and HbI type-specific expression

Q-PCR experiments were performed using the same samples employed in the RT-PCR experiments used for sequencing, and with Q-PCR gene-specific primers (Additional file 4). Large differences were found when expression data from α1 and α2 Hb genes was compared with that observed for the α3 and α4 Hb genes; the former genes exhibited 10 to 100 fold higher expression (Figure 1, Additional file 5). Similar results where found when the expression data for the β1-β4 Hb genes was compared with that of β5. Analysis of data coming from the three HbI (HbI-1/1, HbI-1/2 and HbI-2/2) types showed a strong bias in expression among these HbI types, with 2/2 individuals consistently showing increased expression of α1-α3 and β1-β4 Hb genes relative to the other two HbI types (Figure 1, Additional file 5).

### Abundance of the different types of Hb transcripts in EST databases

We searched the CGP and GenBank databases to gain information on the relative number of ESTs corresponding to the various genes and to the alleles/SNPs identified by our sequencing of the 15 progeny and their parents. The highest number of ESTs was found to match the α1 and α2 Hb genes, followed by β1B and β2 while the lowest number of ESTs was found to correspond to the α4 and β4 Hb genes (Table 2). These results are similar to data obtained by Q-PCR, with the exception of a strong bias in the relative number of ESTs matching the two main alleles identified to be present at the β1 locus: the allele β1B was represented by ten times more ESTs than allele β1A. This difference, however, is likely to be the consequence of a sampling bias, as most fish used to generate EST data in our CGP laboratory had the HbI 2/2 type and, therefore, it is likely that they harboured only the β1B allele. In addition, all the ESTs corresponding to the α4 Hb gene were found to originate from a larval cDNA library, suggesting that this gene is expressed at higher levels at this developmental stage.

**Table 2:** EST abundance in the GenBank database of the nine Hb genes identified in this study.

**Hb gene**	**Polymorphism**	**Number of ESTs**				
		
		**>300**	**200-300**	**20-30**	**10-20**	**<10**
α1	-	+	-	-	-	-
α2	-	+	-	-	-	-
α3	-	-	-	-	+	-
α4	-	-	-	-	-	+
						
β1A	Met55, Lys62	-	-	+	-	-
β1B	Val55, Ala62	-	+	-	-	-
β2	-	-	+	-	-	-
β3	Ser7	-	-	+	-	-
β3	Asn7	-	-	-	-	+
β4	Asp126	-	-	+	-	-
β4	Glu126	-	-	-	-	+
β5	-	-	-	-	-	+

Note: TBLASTN search against GenBank EST_others dataset (*Gadus morhua*, taxid: 8049) was performed using the entire amino acid Hb sequence or with sequences 21 amino acids long: e.g. ten amino acids on each side of the polymorphic site. The search performed with the sequences 21 amino acids long was carried out in order to find ESTs associated with a particular polymorphism.

### Amino acid-based phylogenetic analysis of Atlantic cod hemoglobins

**Figure 2:**
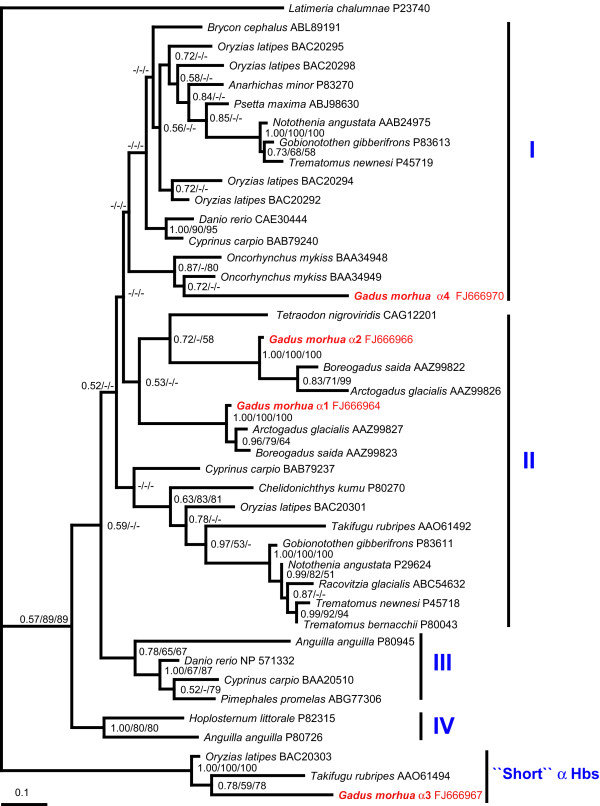
**Phylogenetic Bayesian tree of the α hemoglobins from different teleost fish**. The statistical support for internal nodes was determined by posterior probabilities (Bayesian inference) and bootstrap analyses (Maximum Likelihood and Neighbor-Joining analyses) and is shown, in this order, at the corresponding branches. Values ≤ 50% are indicated by (-). The tree resulting from ML and NJ analysis gave similar topology. The nomenclature of the Hb groups is from Maruyama et al [33].

**Figure 3:**
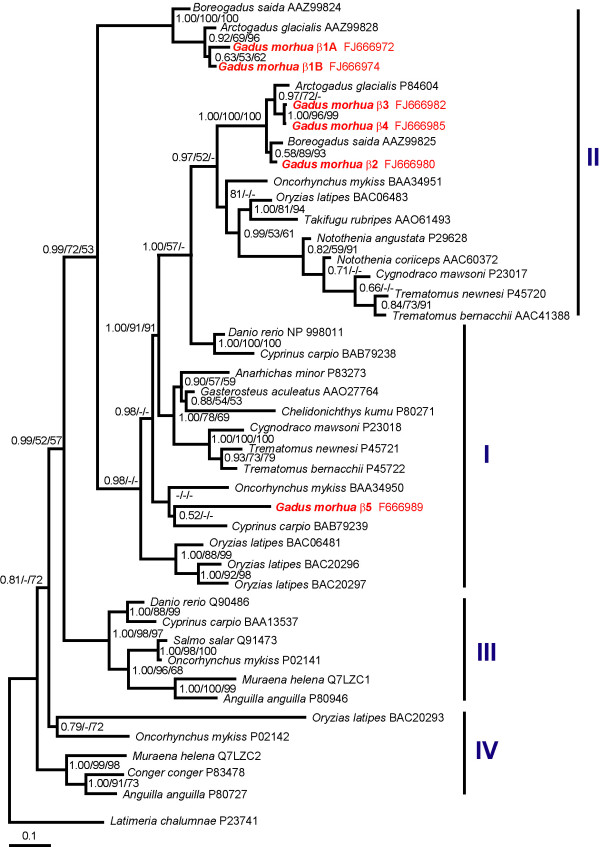
**Phylogenetic Bayesian tree of the β hemoglobins from different teleost fish**. The statistical support for internal nodes was determined by posterior probabilities (Bayesian inference) and bootstrap analyses (Maximum Likelihood and Neighbor-Joining analyses) and is shown, in this order, at the corresponding branches. Values ≤ 50% are indicated by (-). The tree resulting from ML and NJ analysis gave similar topology. The nomenclature of the Hb groups in from Maruyama et al [33].

Phylogenetic analysis of Atlantic cod Hb genes (Additional files 6 and 7) was performed using many of the teleost sequences used in other studies [30,33]; this rationale was chosen in order to maintain the same general tree topology and the main Hb groups [33]. Phylogeny of the α Hbs resolved several clades; with the exception of three sequences that form a strongly supported group which was named "short" α Hbs, these clades were tentatively assigned to the four Hb groups suggested by Maruyama et al [33](Figure 2). The "short" α Hbs group includes the Atlantic cod α3 Hb and consists of α Hbs that encode products that are only 141 amino acids in length. A "short" α Hb sequence was reported previously in adult medaka (*Oryzias latipes*) and was labelled as "unique" [33]. However, this is the first instance where phylogeny revealed that these sequences form a distinct, highly divergent group of fish Hbs. In agreement with their expression pattern, Atlantic cod α1 and α2 Hb sequences clustered with other Hbs that are expressed in adult fish [30], while α4 showed affinities with Hbs demonstrated to be expressed in embryonic stages [34]. Phylogeny of the β Hbs showed that Atlantic cod β1-β4 Hbs have affinities with other β Hbs expressed in adult gadids [30], while Atlantic cod β5 Hb clusters with other embryonic teleost sequences [33,34](Figure 3).

## Discussion

### Gene number and sequence conservation in Atlantic cod hemoglobin

The primers used to amplify the Hb genes were designed based on the data derived from ESTs; therefore, our results are restricted to genes that are expressed in Atlantic cod. We cannot formally rule out that other Hb genes may also be expressed in Atlantic cod; however, taking into account the depth of our EST sequencing from various cDNA libraries, including different developmental stages such as embryonic and larval, the expression of Hb genes not detected here should be extremely low. Further studies, including sequencing of the Hb genomic loci, are required to determine the precise number of Hb genes present in Atlantic cod and their genomic context, and to reveal if, similar to other fish in which these loci are well characterized [27,35], Hb loci in Atlantic cod contain genes that are not expressed (i.e. pseudogenes).

Our study expands the number of Hb transcripts described in Atlantic cod from six [31] to nine, and suggests that the number of Hb heterotetramers present in Atlantic cod, resulting from the combination of the various α and β chains, should be higher than two (the number suggested by agar electrophoresis [7,9,10]) or three (the number suggested recently by Verde and al. [30]). This finding is similar to that of the rainbow trout (*Oncorhynchus mykiss*) Hb system, which was considered for decades to consist of four components [34,36-38]. Similar to Atlantic cod, the electrophoretic or chromatographic separation of rainbow trout Hbs allowed for the identification of several components that can be adult- or embryo/alevin-specific [34]. Functionally, the major fractions HbI and HbIV, which exhibit cathodic and anodic migration upon electrophoretic separation, show radically different oxygen binding properties and pH sensitivity [38-40]. Several studies including crystal structures [36,37] suggested that only one type of alpha and beta chains were present in HbI and HbIV, respectively. However, the separation of trout hemolysate by chromatography followed by amino acid sequencing and mass spectrometry analysis allowed for the identification of nine different alpha and beta chains in these major fractions, and showed that the major rainbow trout Hb components represent a mixture of isoHbs that are made up of more than one alpha and beta chain [41]. Other fish such as carp (*Cyprinus carpio*)[42], goldfish (*Carassius auratus*) [43], medaka [33,35] zebrafish (*Danio rerio*) [44,45] and pufferfish (*Tetraodon nigroviridis*) [46] have multiple α and β genes, and multiple Hb components. Thus, the emerging picture is that the subunit composition of Hbs in many fish is more diverse than previously documented.

### Sequence conservation between β2-β4 genes suggests recent duplications

The search for Hb sequences in fish such as medaka, zebrafish and pufferfish, for which extensive genomic data exists, indicates that these taxa posses multiple α and β genes [33,35,44-46]. In some cases these genes are highly similar in both their coding and non-coding regions; for example the alpha 0 and 1 genes from medaka [33,35]. In most cases however, exons show more sequence conservation than introns, with the 5' and 3' untranscribed regions (UTRs) being responsible for most of the sequence divergence among Hb genes. The exons of β2 and β3-β4 Atlantic cod Hb genes show little differentiation, but both introns and UTR sequences are clearly divergent between genes, with the most marked variation occuring in intron 2, which is 213 nucleotides shorter in β3-β4 Hb genes. When β3 and β4 Hb genes were compared, apart from a small number of SNPs that were detected in exons and introns, but not in the 5' and 3' UTR sequences, and a 4 nt deletion in intron 1 of β4 relative to the β3 gene, no other differences were observed. However, based on the fact that we identified 7 alleles upon sequencing 27 cloned PCR products from parents and 15 progeny (Additional file 3), and on the segregation patterns of the 4 SNPs that were used in the genotyping of more than 1000 fish (our unpublished data), it is clear that at least two different genes encode these sequences. Phylogenetic analysis indicates that the duplication of β2-β4 Hb genes occurred relatively recently within gadids (Figure 3), and suggests that other β genes might be present in Arctic cod (*Arctogadus glacialis*) and polar cod (*Boreogadus saida*), two close relatives of *G. morhua*.

### The β1 Hb gene displays two major allele types correlating with HbI-1 and HbI-2, together with several additional polymorphisms

The β1A and β1B alleles are highly differentiated at both, the nucleotide level (differing by 18 SNPs and multiple indels) and the amino acid level (two amino acids different between β1A alleles and β1B1 and three amino acids between β1A alleles and β1B2). The difference between the two β1A alleles that are expressed affect only 4 sites while 8 sites are variable between the two β1B alleles (Additional file 1). The search of GenBank EST data indicated that while positions 55 and 62 are always linked (i.e. the β1A alleles are always Met55/Lys62 and β1B alleles are Val55/Ala62) the third non-synonymous substitution, Leu123Met can be present in one or the other major allele types. Large-scale sampling of different populations using the GoldenGate genotyping assay that included position 123 also indicated that the non-synonymous substitution Leu123Met is not linked to the polymorphisms present in position 55 and 62 (data not shown). In a paper published at the time this manuscript was drafted, using a SNP assay, Andersen et al [31] analysed the distribution of the substitutions Met55/Lys62 and Val55/Ala62 in Atlantic cod populations from Norwegian coast, Danish waters and western Baltic Sea. Based on statistical analysis they concluded that positions 55 and 62 are strongly linked; in contrast to our approach that comprised whole gene sequencing, their study was restricted to the analysis of these two non-synonymous substitutions that were assayed separately by mass spectrometry [31].

### Is the substitution Lys62Ala unique to Atlantic cod?

**Figure 4:**
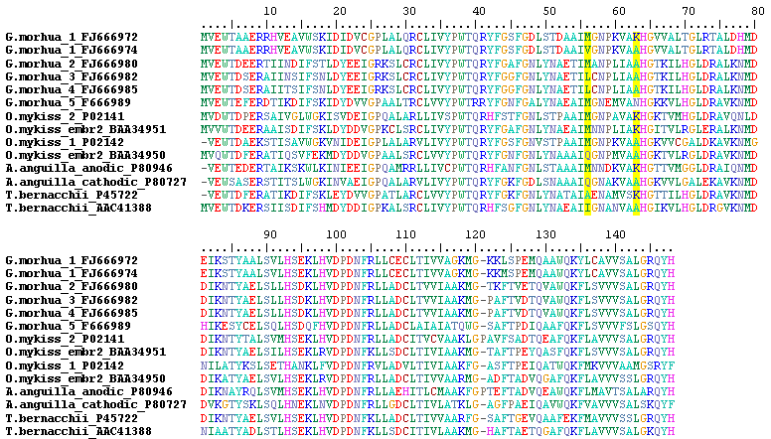
**Alignment of the five Atlantic cod β Hbs and of the β Hbs from rainbow trout (*O. mykiss*), eel (*A. anguilla*) and emerald rockcod (*T. bernacchii*) displaying the substitution Lys62Ala**. In Atlantic cod, the two major alleles β1A and β1B display in position 62 the amino acids Lys (K) and Ala (A), respectively. In the other Atlantic cod Hbs position 62 is occupied by Ala (β2-4 Hbs) or Asn (N; β5 Hb). The other non-conservative substitution identified in the β1 chain of Atlantic cod, Met55Val, seems to have a distribution restricted to this species.

Our finding that the polymorphism at the HbI locus correlates well with the non-conservative substitution Lys62Ala in the β1 Hb gene is further supported by the observation that almost all of the β1 ESTs produced by the CGP correspond to the haplotype β1B (Met55/Lys62) while the Norwegian sequencing project yielded a large number of ESTs matching the haplotype β1A (Val55/Ala62); these results are in agreement with the geographical distribution of the electrophoretic types HbI-2 and HbI-1. Andersen et al. [31] suggested that the substitution Met55Val might have an effect on the stability of the α1β1 heterodimer, while the substitution Lys62Ala might explain the differences in temperature-dependent oxygen affinity documented for HbI-1/1, HbI-1/2 and HbI-2/2 Atlantic cod using isolated erythrocytes or hemolysates. Clearly, the effects of the substitution Met55Val at the αβ interface are hard to predict in the absence of a crystal structure of Atlantic cod Hb, as structure-function analyses of hemoglobins are notoriously difficult [47]. However, the substitution Lys62Ala is located next to the highly conserved His63, which plays a key role in controlling the access of ligands to the heme pocket, and thus is likely to have significant effects. As a more reliable inference cannot be achieved in the absence of a crystal structure for Atlantic cod Hbs, we decided to see if Hbs from other fish display a similar substitution. Indeed, our search in GenBank indicates that other fish, such as rainbow trout, eel (*Anguilla anguilla*), emerald rockcod (*Trematomus bernacchii*) and moray (*Murena helena*) have β Hb genes that display the same polymorphism, Lys/Ala, in position 62 (Figure 4). Notably, while the Lys62Ala substitution in Atlantic cod is related to a genetic system consisting of 2 major alleles/one gene, in rainbow trout these polymorphisms are associated with two genes expressed in juveniles, and with a further two genes expressed in adult fish [34]. Overall, as this substitution is present in other fish it is likely to play a significant role in the biology of Atlantic cod Hbs. However, more studies are needed to determine if any of the additional polymorphisms linked with the β1 Hb alleles can explain why the genotypes described by Hb electrophoresis do not always correlate with phenotypic traits such as acute temperature/hypoxia tolerance [21] or growth characteristics [23,24].

### Concerted changes in the expression of several Hb genes might also be involved in thermal stress/hypoxia responses

Fish having multiple hemoglobin types, such as rainbow trout [39,41,48], carp [42] and goldfish [43], have been shown to alter the relative ratio of anodic to cathodic Hb components over time. This form of acclimation is correlated with changes in temperature, and has been reported in response to seasonal, diurnal, and random temperature cycles [43,48]. In addition, goldfish have the ability to assemble an extra Hb molecule by recombining dimers from existing components [42,43,48]. Given that acclimation of Atlantic cod to different temperatures results in changes in the expression of anodic vs. cathodic components (low temperatures favouring the expression of more anodic hemoglobin components, whereas acclimation to higher temperatures favours the expression of more cathodic/less anodic components [14]), that the ratio of cathodic to anodic hemoglobin components changes during ontogeny [20] and the plethora of Hb genes that are differentially expressed in adult fish of the three hemoglobin genotypes (this study), we suggest that: 1) temperature and hypoxia tolerance may not be entirely dependent on the expression of one or the other major β1 allele types as suggested recently [31]; 2) in the early stages of Atlantic cod development Hb genes other than β1 are likely to be preferentially expressed.

## Conclusion

This study indicates that more Hb genes are present and expressed in adult Atlantic cod than previously documented by using protein gel electrophoresis or protein sequencing. Thus, the emerging picture is that the subunit composition of Atlantic cod Hbs is more diverse than indicated by Verde et al. [30]; this finding suggests that in depth structure-function studies of the various Atlantic cod Hbs are needed to better understand their functional characteristics. The high number of polymorphisms, identified by sequencing the nine Hb genes and their transcripts, suggests that population studies of Atlantic cod employing Hb protein electrophoresis largely underestimated the level of polymorphisms present at Hb loci. Expression data indicates that the major Hb genes expressed in adult fish are α1-α2 and β1-β4 while EST distribution restricted to a larval cDNA library, Q-PCR and phylogenetic analysis suggest that the α4 Hb gene can be regarded as an embryonic/larval Hb that is likely transcribed in larger quantities during early stages of development and at low levels in adult fish; these characteristics also seem to apply to the β5 Hb gene. The function and timing of expression of α3 Hb remains elusive as this gene belongs to an uncharacterized group of fish α Hbs that are unique in encoding only 141 amino acids, two amino acids less than the other α Hbs described in vertebrates.

The high number of Hb genes expressed simultaneously in adult Atlantic cod suggests that the response to environmental challenges such as hypoxia and temperature change might be achieved by modulating their expression. In this context, the relative importance of the non-conservative substitutions Met55Val and Lys62Ala found in β1 Hb gene, which seems to explain the occurrence of the HbI-1 and HbI-2 alleles described by gel electrophoresis, needs to be explored in relation to the many additional Hb genes and alleles, and in regard to expression responses to environmental stimuli. Moreover, the fact that we discovered that fish such as rainbow trout, eel, emerald rockcod, and moray, have β Hb genes that display the same polymorphism, Lys/Ala, in position 62 but which function as part of a different genetic system, i.e. two genes and not two major alleles/one gene as in Atlantic cod, indicate a convergent evolution related to functional characteristics whose importance is waiting to be deciphered.

## Methods

### Identification of Atlantic cod hemoglobin transcripts in databases

The identification of hemoglobin (Hb) genes expressed in Atlantic cod was carried out by analysing the EST data produced by the CGP (see: ), and EST data deposited in GenBank by CGP and other groups (see: ). Identification of Hb transcripts in the CGP database was achieved using keyword searches of data automatically annotated by AutoFact [32] while the search in GenBank was performed by running a BLAST search with Hb sequences retrieved from the CGP database.

### Experimental fish

A single family of Atlantic cod consisting of all three hemoglobin types (HbI-1/1, HbI-2/2 and HbI-1/2) identified using agar gel electrophoresis [7], was produced by crossing heterozygous parents (HbI-1/2)*(HbI-1/2). The progeny were produced by strip spawning parents (wild caught from Cape Sable; 43 20' 04", 65 40 ' 45") at the St. Andrews Biological Station (St. Andrews New Brunswick) in December 2005. Approximately 800 juveniles were transported to the Dr. Joe Brown Aquaculture Research Building (JBARB) at the Ocean Sciences Centre of Memorial University of Newfoundland. At the JBARB, the fish were kept until the beginning of November 2007 when fin clips and blood samples were collected.

### Fin clip DNA isolation and sequencing of Hb genes

Fin clip DNA from five individuals of each hemoglobin type was isolated using the Gentra Puregene DNA isolation procedure (Qiagen, Burlington, ON, Canada). PCR primers were designed based on EST data (Additional file 2). The initial sequencing was performed from PCR amplicons. Good quality sequence data was obtained for the four α Hb genes and for β5, but for β1-β4 Hb genes most chromatograms were of poor quality or unreadable. To overcome this problem, the PCR products of β1-β4 Hb genes were gel purified, extracted using the Qiagen Gel Extraction kit (Qiagen, Burlington, ON, Canada) and cloned in pCR^®^2.1-TOPO^® ^using the TOPO TA cloning kit (Invitrogen, Burlington, ON, Canada). The sequence from two or more clones from each gene and individual was determined using M13 forward and reverse primers. Sequencing was performed at The Atlantic Genome Centre (TAGC, Halifax, NS, Canada) using the MegaBace platform (GE Healthcare Life Sciences, Piscataway, NJ, USA) and at Macrogen (Macrogen USA, Rockville, MD).

### Sample preparation for protein and RNA isolation

Blood samples from 15 individuals (5 from each of the three hemoglobin types) were collected in heparinized tubes and red blood cells (RBCs) were separated from plasma by centrifugation at 3,000 g for 1 minute. Two-thirds of the pelleted RBCs were stored at -80°C and used for protein electrophoresis, while remainder of the pellet was resuspended in RNAlater (Ambion/Applied Biosystems, Austin, TX, USA) at a ratio 1:5 and used for RNA extraction.

### Reverse-transcription PCR (RT-PCR)

Total RNA was extracted from RBCs resuspended in RNAlater using the RNeasy Mini Kit (Qiagen Inc.). RNA quality was assessed by separating samples on 1% native agarose gels. One step RT-PCR was performed using Ambion's RETROscript kit following the manufacturer's instructions. The primers used to distinguish among the various hemoglobin genes are listed in Additional file 2. Sequencing of the RT-PCR products was performed at Macrogen (Macrogen USA, Rockville, MD).

### Quantitative reverse transcription-polymerase chain reaction (Q-PCR)

Expression levels of the nine Hb genes were analysed by quantitative PCR using Fast SYBR Green^® ^and the StepOne Real-Time PCR system (Applied Biosystems, Foster City, CA). For Q-PCR studies, we used 2-3 individuals of each Hb electrophoretic phenotype. The primer sequence used for gene expression analysis is presented in Additional file 4. Primers were first tested by PCR, using genomic DNA to make sure that only one size product was obtained; the amplicons were separated on a 1.5% agarose gel and stained with ethidium bromide. In addition, following Q-PCR amplification, melt-curves (0.3°C from 50°C to 95°C) were used to verify that a single amplicon was obtained and no primer-dimer products were present. Q-PCR was performed in a 20 μl reaction volume using 20 ng of cDNA, 1 uL 4 μM each of forward and reverse primers and 10 uL 2× Fast SYBR Green^® ^Master Mix. Expression levels of hemoglobin genes were normalized to the ubiquitin gene. The decision to use ubiquitin as our endogenous control was based on experimentation we had conducted comparing several housekeeping genes and on supporting literature [49,50]. Cycling parameters consisted of one denaturing cycle of 95°C for 2 min., followed by 40 cycles of 95°C for 30 sec. and 60°C for 1 minute. Cycled 48 well-plates contained target genes and the endogenous control in duplicate. The fluorescence threshold cycle (C_T_) was determined automatically using StepOne software (version 2.0) for comparative C_T _experiments. Transcript abundance was determined using the comparative C_T _method for relative quantification [51], by employing the individuals of a particular HbI phenotype with the lowest gene expression as calibrators. Amplification efficiency (*E *= (10)^(-1/slope)^) was assessed using serial dilutions (1:4); the initial concentration of the template was 20 ng of cDNA. The results were analysed with MiniTab software (version 14, Minitab Inc.). Data were tested for normality using the Anderson-Darling normality test and analysed using one-way ANOVA and Tukey's test.

### Sample preparation for protein electrophoresis

For the protein analysis of hemoglobin phenotypes, 100 μl of red blood cells (RBCs) were washed twice with two volumes of ice-cold saline solution (1 mM Tris, pH 8.0, 200 mM NaCl) and collected by centrifugation at 3,000 × g for 5 min. at 4°C. The RBCs were lysed in 300 μl of cold buffer (1 mM Tris, pH 8.0, 1 mM phenylmethylsulphonyl fluoride). To complete hemolysis, samples were placed on ice for 10 minutes and then at -80°C for 15 min. Cellular debris was pelleted by centrifugation at 15,000 × g for 15 minutes at 4°C and the hemolysate was stored in 100 mM NaCl at -80°C. Protein concentration of the hemolysate was measured using the Bradford method [52].

### Agar gel electrophoresis

For agar gel electrophoresis 20 μl of the hemolysate was mixed with 10 μl of 40% sucrose and 15 μl of the samples were run on a 2% agar gel at 10 V cm^-1 ^for 2.5 to 3 hrs using reverse polarity. Smithies buffer (45 mM Tris, 25 mM boric acid, 1 mM EDTA) pH 8.6, was used as an electrode buffer, and diluted 1:1 with distilled water for the gel buffer. Gels were stained in a Coomassie Blue R-250 solution for 2 hours, and then de-stained overnight in a mixture of 40% methanol:10% acetic acid.

### Isoelectrofocusing (IEF)

All individuals were analysed for the Hb electrophoretic type by IEF to determine if this method could resolve additional polymorphisms relative to those uncovered by agar gel electrophoresis. Protein separation was performed using precast pH 3-10 and pH 5-8 IEF ready gels (Bio-Rad Laboratories, Mississauga, ON, Canada). The cathodic buffer was 20 mM lysine and 20 mM arginine and the anodic buffer was 7 mM phosphoric acid. Gels were run for 2.5 hours using a stepwise protocol: 100 V for 1 hour followed by 250 V for 1 hour and 500 V for 30 minutes. Gels were fixed in 10% trichloroacetic acid and then rinsed in a solution of 40% methanol: 10% acetic acid to remove the ampholytes. Staining was performed as described above for the proteins separated by agar gel electrophoresis. IEF revealed more Hb components than agar electrophoresis but no additional polymorphisms were found within individuals of the three Hb electrophoretic types.

### Phylogenetic analysis

Multiple alignments were done using Clustal X [53] and edited with BioEdit 7.0 [54]. Phylogenetic analyses employed Neighbor-Joining (JTT model, pairwise deletion, bootstrap value 500), Maximum-Likelihood (JTT model, gamma estimated, number of replicates 100), and Bayesian inference (WAG model; 500,000 generations; sample frequency 100; burnin 1000) and were performed using MEGA 4.0 [55], PHYML [56], and Mr. Bayes 3.1 [57], respectively. The phylogenetic trees were displayed using TreeView [58].

## Authors' contributions

TB conceived, designed and implemented all experiments, processed sequencing and Q-PCR data, conducted Hb electrophoresis experiments, performed phylogenetic analyses, interpreted the results and wrote the manuscript; CS conducted sample preparation, cloning, PCR and Q-PCR experiments, interpreted data and was involved in drafting the manuscript; KG initiated studies on Atlantic cod Hb genotypes within the CGP including the production of the fish used in these experiments, provided blood samples of the three Hb electrophoretic phenotypes, and helped with the drafting of the final versions of the manuscript; SB was involved in the design and implementation of the experiments, helped in the interpretation of data and with the final versions of the manuscript. All authors read and approved the final manuscript.

## Supplementary Material

Additional file 1 **Substitution chart of β1 alleles**. This table shows SNP data obtained by analysing the β1 Hb alleles from two heterozygote parents (♀ and ♂, HbI-1/2), and 15 progeny, five individuals for each phenotype. Click here for file

Additional file 2 **Primers used to obtain genomic data for the nine Atlantic cod hemoglobin genes, i.e., α1-4 and β1-5, analysed in this study**. This table lists the primers used for the PCR amplification of the nine hemoglobin genes found in Atlantic cod. Click here for file

Additional file 3 **Substitution chart of β3 and β4 Hb alleles**. This table shows SNP data obtained by analysing the β3 and β4 Hb alleles from two heterozygote parents (♀ and ♂, HbI-1/2), and 15 progeny, five individuals for each phenotype. Click here for file

Additional file 4 **Primers used to analyse the expression of the nine Atlantic cod hemoglobin genes, i.e., α1-4 and β1-5, by quantitative PCR (Q-PCR)**. This table lists the primers used to study by quantitative PCR the expression of the nine hemoglobin genes found in Atlantic cod. Click here for file

Additional file 5 **The expression of the nine Hb genes in individuals of the three HbI electrophoretic types**. This table shows the results obtained by analysing the expression of the nine Hb genes by Q-PCR using the ΔΔCt method and ubiquitin as a reporter gene. Click here for file

Additional file 6 **The deduced amino acid sequence of the four Atlantic cod α Hb genes**. This figure shows the amino acid sequence alignment of the four α Hbs found in Atlantic cod. Click here for file

Additional file 7 **The deduced amino acid sequence of the five Atlantic cod β Hb genes and of their alleles**. This figure shows the amino acid sequence alignment of the five β Hbs found in Atlantic cod and of their alleles. Click here for file
