# Supplementary material for: Atlantic cod (Gadus morhua) hemoglobin genes: multiplicity and polymorphism
Source: BMC Genet. 2009 Sep 3;10:51. doi: 10.1186/1471-2156-10-51 (PMC2757024; doi:10.1186/1471-2156-10-51)
Supplement: Additional file 1 — Substitution chart of β1 alleles. This table shows SNP data obtained by analysing the β1 Hb alleles from two heterozygote parents (♀ and ♂, HbI-1/2), and 15 progeny, five individuals for each phenotype. [file 1471-2156-10-51-S1.doc]

**Additional file 1. Substitution chart of β1 alleles.** Data was obtained by analysing two heterozygote parents (♀ and ♂, HbI-1/2), as determined by analysing their hemoglobin electrophoretic phenotype, and 15 progeny, five individuals for each phenotype (HbI-1/1, HbI-1/2 and HbI-2/2). Data was obtained by sequencing cloned PCR products from both ends; more than one clone was sequenced for parents and most of the progeny. 5’, sequence upstream of the start codon; S, synonymous substitution; N, non-synonymous substitution. The SNPs analysed using the GoldenGate assay are indicated by Roman numerals. ^1^Three polymorphisms were identified for this site; ^2^Indels in intron 2 (+/- nucleotides in alleles A1, A2 and B2 vs allele B1). ^3^TG repeat of ~ 120 bp long. The number of the repeated TG sequence was difficult to asses in some sequencing reactions because of suboptimal signal of the sequencing reaction; in this case the number was modified to match the average length determined for that particular allele.

|  | Exons/introns | **5’** | **E** | **I** | **I** | **I** | **I** | **I** | **E** | **E** | **E** | **E** | **E** | **I** | **I** | **I** | **I** | **I** | **E** |
| --- | --- | --- | --- | --- | --- | --- | --- | --- | --- | --- | --- | --- | --- | --- | --- | --- | --- | --- | --- |
|  | Position of the substitutions | T/A  -8 | T/C  21 | G/A  115 | G/A  143 | A/G  166 | G/A  227 | -/G  /T  233^1^ | G/A  432 | T/C  452 | G/A  453 | C/A  454 | G/A  473 | 599-649^2^ | C/T  731 | A/T  740 | 828-834^2^ | 906-1033^3^ | A/T  1092 |
|  | Substitution type |  | **S** |  |  |  |  |  | **N** | **S** | **N** | **-** | **S** |  |  |  |  |  | **N** |
|  | Amino acid change |  |  |  |  |  |  |  | **Met/Val** |  | **Lys/Ala** |  |  |  |  |  |  |  | **Leu/Met** |
|  | SNPs genotyped using the GoldenGate assay |  | **I** |  |  |  |  |  |  |  |  |  |  |  |  | **II** |  |  | **III** |
| **Alleles** | **# of progeny (# of sequenced clones); parents (# of sequenced clones)** |  |  |  |  |  |  |  |  |  |  |  |  |  |  |  |  |  |  |
|  |  |  |  |  |  |  |  |  |  |  |  |  |  |  |  |  |  |  |  |
| **A1** | 7 (10); ♀ (2) | **T** | **C** | **A** | **G** | **G** | **A** | **G** | **A** | **C** | **A** | **A** | **G** | **+5** | **T** | **T** | **-7** |  | **T** |
| **A2** | 4 (7); ♂(2) | **A** | **C** | **A** | **G** | **G** | **A** | **T** | **A** | **C** | **A** | **A** | **A** | **-7** | **C** | **T** | **-7** | **+14** | **T** |
|  |  |  |  |  |  |  |  |  |  |  |  |  |  |  |  |  |  |  |  |
| **B1** | 5 (6); ♀ (2) | **T** | **T** | **G** | **G** | **A** | **G** | **-** | **G** | **T** | **G** | **C** | **G** |  | **C** | **A** |  |  | **A** |
| **B2** | 3 (3); ♂(2) | **T** | **T** | **A** | **A** | **A** | **A** | **T** | **G** | **T** | **G** | **C** | **G** | **+8** | **T** | **A** |  | **+26** | **T** |
|  |  |  |  |  |  |  |  |  |  |  |  |  |  |  |  |  |  |  |  |
| **B2/B1** | Recombinant 2 (2) | T | T | A | A | A | A | T | G | T | G | C | G |  | C | A |  |  |  |
| **B2/A2** | Recombinant ♂ (1) | T | T | A | A | A | A | T | A | C | A | A | A |  | C | T | -7 | +14 | T |
| **B1/A1/B1** | Recombinant ♀ (2) | T | T | G | G | A | G | - | A | C | A | A | G |  | C | A |  |  | A |
| **B1/A1** | Recombinant ♀ (2) | T | T | G | G | A | G | - | G | T | G | C | G |  | T | T | -7 |  | T |
