# Supplementary material for: Atlantic cod (Gadus morhua) hemoglobin genes: multiplicity and polymorphism
Source: BMC Genet. 2009 Sep 3;10:51. doi: 10.1186/1471-2156-10-51 (PMC2757024; doi:10.1186/1471-2156-10-51)
Supplement: Additional file 2 — Primers used to obtain genomic data for the nine Atlantic cod hemoglobin genes, i.e., α1-4 and β1-5, analysed in this study. This table lists the primers used for the PCR amplification of the nine hemoglobin genes found in Atlantic cod. [file 1471-2156-10-51-S2.doc]

**Additional file 2. Primers used to obtain genomic data for the nine Atlantic cod hemoglobin genes, i.e., α1-4 and β1-5.**

| Primer name | Sequence (5’ to 3’) |
| --- | --- |
| α1F  α1R  α2F  α2R  α3F  α3R  α4F  α4R  β1F  β1R  β2F  β2R  β3F/β4F  β3R/β4R  β5F  β5R | TATTGAACTGTGCCACAGAGTCCAATCTG  CCTGATACTTTCATCCATCCTATTGTC  GAGCATCAGCAACTTGTTCTTTCAGTCTAG  CATGCATCAATGATGGCGGGAGTCTTC  TGGATATTCAATTGGGGCCACATTTG  CGACTTCATTGGATGAGCAACATCGG  GCCTACAACATGAGTCTCACAGAC  CATGTGCCGATGATGACCATTTCGC  CTTGAGATTCAGCCTAAGCTACATTGAACC  GGGTTTGTCACTGAAGTTGTAGCTTGCTG  GGACGATTCAGTTTGATTTTCTACTGAAGC  GATCGGTGATGATGAATGGGATGGCTGG  CACATCAGCAACCATGGTTGAGTGGAC  CAAGAAAATCTGCATTTTGTTGAATATCAGC  AGCGGAAACAATCACCACAGCCG  GTTGATGTTGTTGTTCACGGTAGTCC |
