# Supplementary material for: Atlantic cod (Gadus morhua) hemoglobin genes: multiplicity and polymorphism
Source: BMC Genet. 2009 Sep 3;10:51. doi: 10.1186/1471-2156-10-51 (PMC2757024; doi:10.1186/1471-2156-10-51)
Supplement: Additional file 3 — Substitution chart of β3 and β4 Hb alleles. This table shows SNP data obtained by analysing the β3 and β4 Hb alleles from two heterozygote parents (♀ and ♂, HbI-1/2), and 15 progeny, five individuals for each phenotype. [file 1471-2156-10-51-S3.doc]

**Additional file 3. Substitution chart of β3 and β4 Hb alleles.** Data was obtained by analysing two heterozygote parents (♀ and ♂, HbI-1/2), as determined by analysing their hemoglobin electrophoretic phenotype, and 15 progeny, five individuals for each phenotype (HbI-1/1, HbI-1/2 and HbI-2/2). Data was obtained by sequencing cloned PCR products from both ends; more than one clone was sequenced for parents. S, synonymous substitution; N, non-synonymous substitution. The SNPs analysed using the GoldenGate assay are indicated by Roman numerals. Analysis of SNP I using the GoldenGate assay revealed that all individuals appear to be heterozygous, including both the parents and all progeny from 3 Atlantic cod families. We have found this to be diagnostic of putative SNPs which are, in fact, variants of 2 different genes rather than different alleles of a single gene. Therefore, this is a clear indication that two genes, β3 and β4, are present in Atlantic cod. ^1^Indel present in intron 1. Many sequences deposited in GenBank from samples collected in Icelandic waters (GenBank # EF644855-EF644912) have a SNP profile similar to that of the β4 Hb alleles.

|  | Exons/introns | **E** | **E** | **I** | **I** | **I** | **E** | **E** | **I** | **E** | **E** | **E** | **E** | **E** |
| --- | --- | --- | --- | --- | --- | --- | --- | --- | --- | --- | --- | --- | --- | --- |
|  | Position of the substitutions | G/A  20 | A/C  38 | A/C  95 | T/C  102 | 128-131^1^ | C/T  356 | C/A  419 | A/G  472 | C/T  594 | T/G  609 | C/G  612 | G/C  657 | G/A  666 |
|  | Substitution type | **N** | **N** |  |  |  | **S** | **S** |  | **S** | **N** | **S** | **S** | **S** |
|  | Amino acid change | **Ser/Asn** | **Asn/Thr** |  |  |  |  |  |  |  | **Asp/Glu** |  |  |  |
|  | SNPs genotyped using the GoldenGate assay |  |  |  | **I** |  | **II** | **III** |  |  |  |  | **IV** |  |
| **Gene/**  **Alleles** | **# of progeny (# of sequenced clones); parents (# of sequenced clones)** |  |  |  |  |  |  |  |  |  |  |  |  |  |
| **β 3** |  |  |  |  |  |  |  |  |  |  |  |  |  |  |
| **Allele 1** | 4(4) ♂ (2) ♀(1) | **G** | **A** | **A** | **T** |  | **C** | **C** | **A** | **C** | **T** | **C** | **G** | **G** |
| **Allele 2** | 4(4) | **G** | **A** | **A** | **T** |  | **C** | **C** | **G** | **C** | **T** | **C** | **C** | **G** |
| **Allele 3** | 1 (1) ♂ (2) | **A** | **A** | **A** | **T** |  | **C** | **C** | **A** | **C** | **T** | **C** | **G** | **G** |
| **β4** |  |  |  |  |  |  |  |  |  |  |  |  |  |  |
| **Allele 1** | 5 (5), ♂ (2) | **G** | **C** | **C** | **C** | **-4** | **C** | **C** | **A** | **C** | **T** | **C** | **G** | **G** |
| **Allele 2** | 1 (1), ♀(1) | **G** | **C** | **C** | **C** | **-4** | **T** | **C** | **A** | **C** | **T** | **C** | **G** | **G** |
| **Allele 3** | 2(2) | **G** | **C** | **C** | **C** | **-4** | **C** | **A** | **A** | **C** | **T** | **C** | **G** | **G** |
| **Allele 4** | 1 (1) ♀(1) | **G** | **C** | **C** | **C** | **-4** | **T** | **C** | **A** | **T** | **G** | **G** | **G** | **A** |
