# Supplementary material for: Atlantic cod (Gadus morhua) hemoglobin genes: multiplicity and polymorphism
Source: BMC Genet. 2009 Sep 3;10:51. doi: 10.1186/1471-2156-10-51 (PMC2757024; doi:10.1186/1471-2156-10-51)
Supplement: Additional file 4 — Primers used to analyse the expression of the nine Atlantic cod hemoglobin genes, i.e., α1-4 and β1-5, by quantitative PCR (Q-PCR). This table lists the primers used to study by quantitative PCR the expression of the nine hemoglobin genes found in Atlantic cod. [file 1471-2156-10-51-S4.doc]

**Additional file 4. Primers used to analyse the expression of the nine Atlantic cod hemoglobin genes, i.e., α1-4 and β1-5, by quantitative PCR (Q-PCR).**

| Hemoglobin genes | Primer name | Sequence (5’ to 3’) | Efficiency % | Amplicon size |
| --- | --- | --- | --- | --- |
| α1 | A1SF | GACTTACTTCAGCCACTGGAAGAGCCT C | 82 | 153 |
|  | A1SR | TTGAAGGCGTGCAGCTCGCTCAGAG |  |  |
| α2 | A2SF | GTCCTATTTCTCTCACTGGAAGGACGCG | 82 | 153 |
|  | A2SR | ATGAACGCGTGCAGCTCGCTAAGGC |  |  |
| α3 | A3F | CACATCATACCCTGGCACCAAGAC | 92 | 172 |
|  | A3R | CTGGTAGGCGTGGTAGGTTTGAAGAG |  |  |
| α4 | A4F | TTCTCCCACTGGAAAGACCTCGG | 90 | 138 |
|  | A4R | ATGGAGCTCACTGAGCTCGAGAAG |  |  |
|  |  |  |  |  |
|  |  |  |  |  |
| β1 allele 1 | B1AF | TTATGGGAAACCCCAAGGTGGCCAA | 90 | 131 |
|  | B1SR | GTGCAGTTTCTCGGAGTGCAGCACGC |  |  |
| β1 allele 2 | B1BF | TTGTGGGAAACCCCAAGGTGGCTGC | 99 | 131 |
|  | B1SR | GTGCAGTTTCTCGGAGTGCAGCACGC |  |  |
| β2 | B21CF | CCTGTACAATGCAGAGACCATCATGGC | 82 | 151 |
|  | B2SR | GTGCAGCTTGTCAGAGTGGAGCAGAG |  |  |
| β3 | B3F | ACAGATAGTGAGCGCGCCATCATTAA | 93 | 176 |
|  | B34R | GCGGCGATCAGGGGGTTGCACAG |  |  |
| β4 | B4F | ACAGATAGTGAGCGCGCCATCATTAC | 93 | 176 |
|  | B34R | GCGGCGATCAGGGGGTTGCACAG |  |  |
| β5 | B5F | GTGGACTCGGAGGTACTTTGGAAAC | 87 | 168 |
|  | B5R | TGCAGCTGACTGAGCTCGCAATAG |  |  |
