# Supplementary material for: Atlantic cod (Gadus morhua) hemoglobin genes: multiplicity and polymorphism
Source: BMC Genet. 2009 Sep 3;10:51. doi: 10.1186/1471-2156-10-51 (PMC2757024; doi:10.1186/1471-2156-10-51)
Supplement: Additional file 5 — The expression of the nine Hb genes in individuals of the three HbI electrophoretic types. This table shows the results obtained by analysing the expression of the nine Hb genes by Q-PCR using the ΔΔCt method and ubiquitin as a reporter gene. [file 1471-2156-10-51-S5.doc]

**Additional file 5. The expression of the nine Hb genes in individuals of the three HbI electrophoretic types.** Q-PCR analysis was performed using the ΔΔCt method and ubiquitin as a reporter gene. Data for every gene and HbI type was normalized in two different ways: fold increase was estimated relative to the gene and HbI type with the lowest expression, i.e. the α4 Hb gene, the electrophoretic type HbI-2/2 (column 3), or for every Hb gene and HbI type relative to the HbI type with the lowest expression (column 5). Fold difference was calculated taking into account the values of efficiency of amplification that are listed in Additional file 4.

| Hb genes or alleles for β1 | HbI electrophoretic type | Fold difference relative to the gene and HbI type with the lowest expression (α4; HbI-2/2) | Differences in expression that are statistically significant relative to the HbI type | Fold difference for every Hb gene relative to the HbI type that showed the lowest expression |
| --- | --- | --- | --- | --- |
| α1 | 1/1 | 497.7±135.2 |  | 1.4 |
|  | 1/2 | 353.8±140.5 |  | 1 |
|  | 2/2 | 1311.8±254.1 | p<0.000 | 3.7 |
| α2 | 1/1 | 467.5±153.7 |  | 1.3 |
|  | 1/2 | 358.4±68 |  | 1 |
|  | 2/2 | 1256.1±309 | p<0.000 | 3.5 |
| α3 | 1/1 | 7.4±0.8 |  | 1 |
|  | 1/2 | 21±3.6 |  | 2.8 |
|  | 2/2 | 34.4±12.6 | p<0.002 | 4.6 |
| α4 | 1/1 | 3±1.1 |  | 3 |
|  | 1/2 | 6.1±0.9 |  | 6.1 |
|  | 2/2 | 1 | p<0.000 | 1 |
| β1A | 1/1 | 1205.9±268 |  | 1.6 |
|  | 1/2 | 735.6±207.6 |  | 1 |
| β1B | 1/2 | 395.6±263.7 |  | 1 |
|  | 2/2 | 2620±799.8 | p<0.000 | 6.6 |
| β2 | 1/1 | 333.5±92.4 |  | 1.3 |
|  | 1/2 | 249.8±99 |  | 1 |
|  | 2/2 | 1945.9±653.6 | p<0.000 | 7.8 |
| β3 | 1/1 | 216.2±56.5 |  | 1.2 |
|  | 1/2 | 176.5±62.3 |  | 1 |
|  | 2/2 | 871.1±305.9 | p<0.001 | 4.9 |
| β4 | 1/1 | 146.2±28.3 |  | 1.1 |
|  | 1/2 | 132.6±56.8 |  | 1 |
|  | 2/2 | 533.5±237.8 | p<0.005 | 4 |
| β5 | 1/1 | 3.1±0.9 | p<0.001 | 1 |
|  | 1/2 | 9.2±0.7 |  | 3 |
|  | 2/2 | 14.5±4.7 |  | 4.7 |
