# Supplementary material for: Atlantic cod (Gadus morhua) hemoglobin genes: multiplicity and polymorphism
Source: BMC Genet. 2009 Sep 3;10:51. doi: 10.1186/1471-2156-10-51 (PMC2757024; doi:10.1186/1471-2156-10-51)
Supplement: Additional file 6 — The deduced amino acid sequence of the four Atlantic cod α Hb genes. This figure shows the amino acid sequence alignment of the four α Hbs found in Atlantic cod. [file 1471-2156-10-51-S6.doc]

**Additional file 6. The deduced amino acid sequence of the four Atlantic cod α Hb genes.**

1 80

Hbα1 MSLTPKDKAT VKLFWGRMSG KAELIGADAL SRMLAVYPQT KTYFSHWKSL SPGSPDVKKH GKTIMMGIGD AVTKMDDLER

Hbα2 MSLSSKQKAT VKDFFSKMST RSDDIGAEAL SRLVAVYPQT KSYFSHWKDA SPGSAPVRKH GITIMGGVYD AVGKIDDLKG

Hbα4 MSLTDKDKAL IKGFFAKVSS KAVEIGHQTL ARTIVVYPQT KVYFSHWKDL GPDSPNIRKH GYTVVKGVLD SVDLIDDLVG

Hbα3 MLSKQEKEL IIEIWTRLTP LADRIGAEAL LRMFTSYPGT KTYFSHL-DI TPRSAHLLSH GQKIFLALAE GSKDIANLMT

81 143

Hbα1 GLLTLSELHA FKLRVDPTNF KLLSLNILVV MAIMFPDDFT PMAHLAVDKF LCALALALSE KYR

Hbα2 GLLSLSELHA FMLRVDPVNF KLLAHCMLVC MSMIFPEEFT PQVHVAVDKF LAQLALALAE KYR

Hbα4 GLLELSELHA FRLRIDPANF KILNLNLVVV LGLMFPDDFT PQVHVSVDKY LALICLALCE KYR

Hbα3 NLAPLQTYHA YQLRIQPNNF KLFSHCMIVT LACFMGDRFT PSSHAAMDKY LSAFSAVPGE KFR
