# Supplementary material for: Atlantic cod (Gadus morhua) hemoglobin genes: multiplicity and polymorphism
Source: BMC Genet. 2009 Sep 3;10:51. doi: 10.1186/1471-2156-10-51 (PMC2757024; doi:10.1186/1471-2156-10-51)
Supplement: Additional file 7 — The deduced amino acid sequence of the five Atlantic cod β Hb genes and of their alleles. This figure shows the amino acid sequence alignment of the five β Hbs found in Atlantic cod and of their alleles. [file 1471-2156-10-51-S7.doc]

**Additional file 7. The deduced amino acid sequence of the five Atlantic cod β Hb genes and of their alleles.** Highlighted in yellow are the three non-synonymous substitutions (Met55Val, Lys62Ala and Leu123Met) present in the Hbβ1 alleles.

1 80

Hbβ1A1-2 MVEWTAAERR HVEAVWSKID IDVCGPLALQ RCLIVYPWTQ RYFGSFGDLS TDAAIMGNPK VAKHGVVALT GLRTALDHMD

Hbβ1A1 MVEWTAAERR HVEAVWSKID IDVCGPLALQ RCLIVYPWTQ RYFGSFGDLS TDAAIVGNPK VAAHGVVALT GLRTALDHMD

Hbβ1B2 MVEWTAAERR HVEAVWSKID IDVCGPLALQ RCLIVYPWTQ RYFGSFGDLS TDAAIVGNPK VAAHGVVALT GLRTALDHMD

Hbβ2 MVEWTDEERT IINDIFSTLD YEEIGRKSLC RCLIVYPWTQ RYFGAFGNLY NAETIMANPL IAAHGTKILH GLDRALKNMD

Hbβ3_1-2 MVEWTDSERA IINSIFSNLD YEEIGRKSLC RCLIVYPWTQ RYFGGFGNLY NAETILCNPL IAAHGTKILH GLDRALKNMD

Hbβ3_3 MVEWTDNERA IINSIFSNLD YEEIGRKSLC RCLIVYPWTQ RYFGGFGNLY NAETILCNPL IAAHGTKILH GLDRALKNMD

Hbβ4_1-3 MVEWTDSERA IITSIFSNLD YEEIGRKSLC RCLIVYPWTQ RYFGGFGNLY NAETILCNPL IAAHGTKILH GLDRALKNMD

Hbβ4_4 MVEWTDSERA IITSIFSNLD YEEIGRKSLC RCLIVYPWTQ RYFGGFGNLY NAETILCNPL IAAHGTKILH GLDRALKNMD

Hbβ5 MVEWTEFERD TIKDIFSKID YDVVGPAALT RCLVVYPWTR RYFGNFGALY NAEAIMGNEM VANHGKKVLH GLDRAVKNMD

81 147

Hbβ1A1-2 EIKSTYAALS VLHSEKLHVD PDNFRLLCEC LTIVVAGKMG KKLSPEMQAA WQKYLCAVVS ALGRQYH

Hbβ1B1 EIKSTYAALS VLHSEKLHVD PDNFRLLCEC LTIVVAGKMG KKMSPEMQAA WQKYLCAVVS ALGRQYH

Hbβ1B2 EIKSTYAALS VLHSEKLHVD PDNFRLLCEC LTIVVAGKMG KKLSPEMQAA WQKYLCAVVS ALGRQYH

Hbβ2 DIKNTYAELS LLHSDKLHVD PDNFRLLADC LTVVIAAKMG TKFTVETQVA WQKFLSVVVS ALGRQYH

Hbβ3_1-2 DIKNTYAELS LLHSDKLHVD PDNFRLLADC LTVVIAAKMG PAFTVDTQVA WQKFLSVVVS ALGRQYH

Hbβ3_3 DIKNTYAELS LLHSDKLHVD PDNFRLLADC LTVVIAAKMG PAFTVDTQVA WQKFLSVVVS ALGRQYH

Hbβ4_1-3 DIKNTYAELS LLHSDKLHVD PDNFRLLADC LTVVIAAKMG PAFTVDTQVA WQKFLSVVVS ALGRQYH

Hbβ4_4 DIKNTYAELS LLHSDKLHVD PDNFRLLADC LTVVIAAKMG PAFTVETQVA WQKFLSVVVS ALGRQYH

Hbβ5 HIKESYCELS QLHSDQFHVD PDNFRLLADC LAIAIATQWG SAFTPDIQAA FQKFLSVVVF SLGSQYH
